# Accelerating digital innovation in clinical neuropsychology: simulation approach to support medical device certification

**DOI:** 10.3389/fdgth.2025.1646694

**Published:** 2026-02-12

**Authors:** Andrea Panzavolta, Federico Sternini, Paolo Caffarra, Dalila De Vita, Alessandra Dodich, Cristina Fonti, Federica L’Abbate, Luigi Lavorgna, Valentina Laganà, Camillo Marra, Costanza Papagno, Francesca Ferrari Pellegrini, Andrea Stracciari, Luigi Trojano, Tiziana Iaquinta, Roberta Pandolfi, Monica Calore, Sveva Sanzone, Alice Ravizza, Stefano F. Cappa, Chiara Cerami

**Affiliations:** 1 IUSS Cognitive Neuroscience (ICoN) Center, Scuola Universitaria di Studi Superiori IUSS, Pavia, Italy; 2 InsideAI srl, I3P Politecnico di Torino, Torino, Italy; 3 PolitoBIOMed Lab, Politecnico di Torino, Torino, Italy; 4 Membro Esperto Tavolo Permanente Sulle Demenze del Ministero Della Salute, Rome, Italy; 5 Department of Psychology, University of Campania “Luigi Vanvitelli”, Caserta, Italy; 6 Center for Mind/Brain Sciences (CIMeC), University of Trento, Rovereto, Italy; 7 IRCCS, Istituto Delle Scienze Neurologiche di Bologna, Bologna, Italy; 8 Neurology Unit, Fondazione Policlinico Universitario “A. Gemelli” IRCCS, Rome, Italy; 9 Clinica NeurologicaI, Azienda Ospedaliera Universitaria, Università Della Campania “Luigi Vanvitelli”, Naples, Italy; 10 Associazione per la Ricerca Neurogenetica odv, Lamezia Terme, Italy; 11 Università Cattolica del Sacro Cuore, Rome, Italy; 12 Geriatric Clinic Unit, Medical Geriatric Rehabilitative Department, University Hospital of Parma, Parma, Italy; 13 Xenia Reply, Milan, Italy; 14 Biogen Italy, Milan, Italy; 15 Department of Neurology and Laboratory of Neuroscience, IRCCS Istituto Auxologico Italiano, Milano, Italy; 16 Istituti Clinici Scientifici Maugeri IRCCS, Brain e-Health Aging (BeA) Laboratory, Department of Neurorehabilitation, Milan, Italy

**Keywords:** Alzheimer, mild cognitive impairment, digital health, teleneuropsychology, SaMD

## Abstract

**Background:**

In recent years, the focus on digitization of neuropsychological procedures in memory clinics has become paramount. Several teleneuropsychology platforms have been developed for testing patients with cognitive deficits, but only a few have been registered as medical devices (MD) being available in clinical practice. Hereby, we present a simulation-based novel approach designed to test technical performance and provide pre-clinical validation of a novel teleneuropsychology platform (i.e., Tenèpsia®) as required for the certification of Software as a Medical Device (SaMD) under the European regulation 2017/745 (MDR).

**Methods:**

Six dummy cognitive profiles simulating virtual patients with different cognitive performances were created. Five internal and two external experts evaluated simulated performances for representativeness, coherence and credibility.

**Results:**

One cognitively unimpaired and five mild cognitive impairment (MCI) profiles were considered. Demographic features and target cognitive scores were derived by reference literature for each simulated user. Representativeness was rated as more than 75% accurate by internal and external experts. Coherence and credibility were considered adequate to support SaMD certification.

**Conclusions:**

Certification of digital solutions as SaMD may require costly and time-consuming validation procedures. Simulation-based approach based on synthetic data is a valid method to overcome this limitation, that can be easily implemented to test platform, accelerating innovation in teleneuropsychology and providing adequate evidence of safety, efficacy and long-term comparability with the standard of care.

## 1 Introduction

Prevention, early diagnosis and management of cognitive disorders are among the greatest global health and social care challenges of our time (1) representing a crucial priority. Cognitive assessment with harmonized neuropsychological testing is the gateway in the diagnostic roadmap for patients suspected of cognitive decline, needed for a correct definition of the syndromic presentation and to correctly address the pathway to biological diagnosis (2–4). Nonetheless, the use of harmonized cognitive testing in clinics is puzzled due to lack of time and resources, and of specific expertise both in primary care and in specialised settings (5, 6).

Digital solutions may offer the opportunity to overcome current limitations in clinical neuropsychology by measuring a broad range of cognitive abilities remotely and with relevant time-saving and reduced resources. Although the pandemic has amplified the offer of telemedicine services, harmonized and clinically validated teleneuropsychology batteries on digital solutions are still lacking. Crucially, there are too few software tools certified for this purpose in Europe [see for example Lesoil et al. (7)]. One of the main obstacles is indeed obtaining the software as medical device (SaMD) certification according to the European regulation 2017/745 (MDR). The procedure requires to prove that the risk-benefit profile of the device is non-inferior to the currently available state of the art. This may be done by performing a comparison with the standard of care. However, this approach is often expensive and time-consuming. Performance simulations are thus increasingly used in digital health to prove SaMD reliability, reproducibility, and safety prior to clinical validation, ensuring that simulated performance does not materially diverge from real-world performance, accelerating digital innovation and providing better resilience to privacy attack and a statistical validity comparable to the traditional approach (8).

In this study, we describe the methodological approach adopted for the analytical/technical and pre-clinical validation of a novel teleneuropsychology platform [i.e., Tenèpsia® (9)], already tested for usability and user-friendless (10), to prove its technical performance and suitability for EU certification under the 2017/745 MDR.

## 2 Materials and methods

### 2.1 Creation of dummy users, simulation and evaluation of performance

In the first phase, we created dummy cognitive profiles and simulated performances of potential users of the digital platform to verify that the system operates as intended, before exposure to real patients or acquisition of clinical data and according to the early-stage pre-clinical requirements of SaMD. Five internal experts from the Tenèpsia® working group (A.P., C.C., C.M., P.C., & S.C.) met twice a week for two months in a focus group to define dummy user profiles. These meetings involved both clinicians and software engineers, with the aim of jointly defining the expected cognitive patterns and translating them into simulated test scores based on literature and normative datasets. Since the Tenèpsia® platform was designed to test individuals with suspected mild cognitive impairment (MCI) (9), the relevant literature was considered as reference evidence [see Petersen et al. (11) for a review]. Average age of dummy users was derived from literature evidence (12) and average education from the last report of Italian National Institute of Statistics [i.e., ISTAT (13)]. Simulated cognitive performances of dummy users were defined based on Italian normative data and error profiles reported in literature (14–26). Mean and standard deviation for each neuropsychological test included in the platform (9) were considered as reference standards. In accordance with the international guidelines for neuropsychological testing (27), impaired scores in MCI were simulated by considering a performance deviating two standard deviations from the mean score of cognitive unimpaired subjects. For those digital tests in which the number of items differed from the paper-and-pencil version [see Panzavolta et al. (9), for details], mean and standard deviation scores were normalized. After the definition of demographic features and simulated test scores of dummy profiles, the scenarios were run on the Tenèpsia® platform through automated scripts developed by the engineering team based on the target cognitive profiles identified by clinical experts. These scripts interacted with the platform as real users (i.e., by generating plausible responses for each test on the platform). Simulated performances were thus directly executed by the software enabling to verify the correct generation of the expected cognitive outputs on the platform.

In the second phase, dummy user profiles were evaluated for representativeness, defined here as the extent to which the simulations accurately reflect user performances in a real-world context, coherence and credibility of the simulated cognitive profiles. Two experts in clinical neuropsychology external from the Tenèpsia® working group were involved for the evaluation phase to ensure the reliability of the simulations and to meet the standards required for EU SaMD certification (28). Both internal and external experts completed the evaluations for each dummy user. Data were collected through an *ad hoc* questionnaire implemented on Google Forms® (see Supplementary Table S1). Percentage on accuracy in representativeness and average credibility and coherence scores were computed. Upon conclusion of the evaluation of each dummy user, A.P. and F.S. conducted qualitative interviews to understand the reasons for the choices made through the questionnaire.

## 3 Results

### 3.1 Dummy user profiles

At the end of the focus group activities, internal experts agreed on the definition of six dummy users: one with a normal cognitive profile and five with different MCI profiles. This final set of six dummy users was selected to ensure a full coverage of the cognitive profiles relevant for the intended use of the teleneuropsychology platform (i.e., the early detection of mild neurocognitive disorder, namely MCI) (11). Since our goal was the technical validation of the platform as required for MDR pre-clinical assessment, we included the minimum core set of cognitive profiles more frequently reported in literature (11) to test the platform across distinct MCI patterns without redundancy and ensuring feasibility for expert-based evaluations. The MCI dummy user profiles included: one amnestic single-domain MCI (aMCI-sd) with long-term episodic memory impairments only, three amnestic multi-domain MCI (aMCI-md) with memory plus executive/attention or language or visuo-spatial impairments and one non-amnestic single-domain (naMCI-sd) with executive/attention impairments only. Literature search identified an average age of 73.4 years for MCI individuals. According to this average age, the reference education level identified by the 2020 ISTAT report was the degree of primary school (i.e., 5 years). Reference normative data for each test included in the Tenèpsia® platform were thus selected considering means and standard deviations of individuals of an age of 73 years and ≤5 years of education (see Supplementary Table S2 for simulated test scores).

### 3.2 Representativeness, coherence and credibility

**Figure 1:**
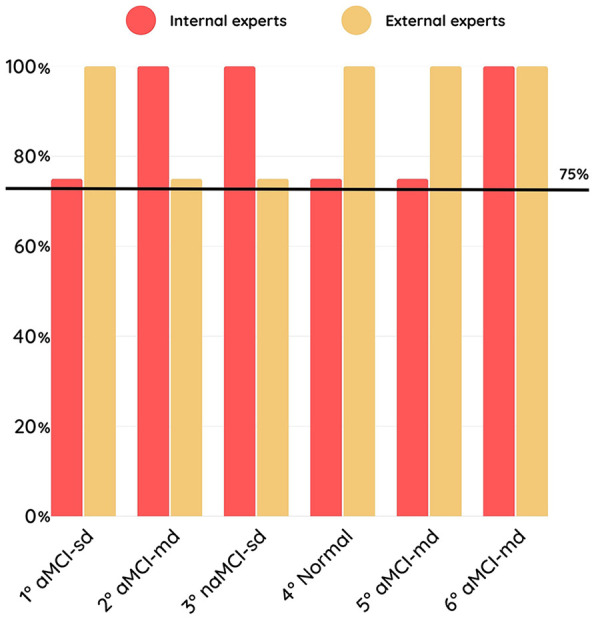
Percentage accuracy in correctly identifying the representativeness of each dummy user profile. The y-axis reports the overall accuracy (%) in recognizing the cognitive profile of each dummy user. The x-axis shows the different dummy user profiles: 1° aMCI-sd, single-domain amnestic Mild Cognitive Impairment; 2° aMCI-md, multi-domain amnestic Mild Cognitive Impairment, memory plus executive/attention; 3° naMCI-sd, single-domain non-amnestic Mild Cognitive Impairment, executive/attention; 4° Normal, cognitively unimpaired profile; 5° naMCI-md, multi-domain non-amnestic Mild Cognitive Impairment, memory plus language; 6°naMCI-md, multi-domain non-amnestic Mild Cognitive Impairment, memory plus visuo-spatial. Accuracy values are reported separately for internal experts and external experts. The horizontal dashed line indicates the predefined acceptability threshold (75%).

**Figure 2:**
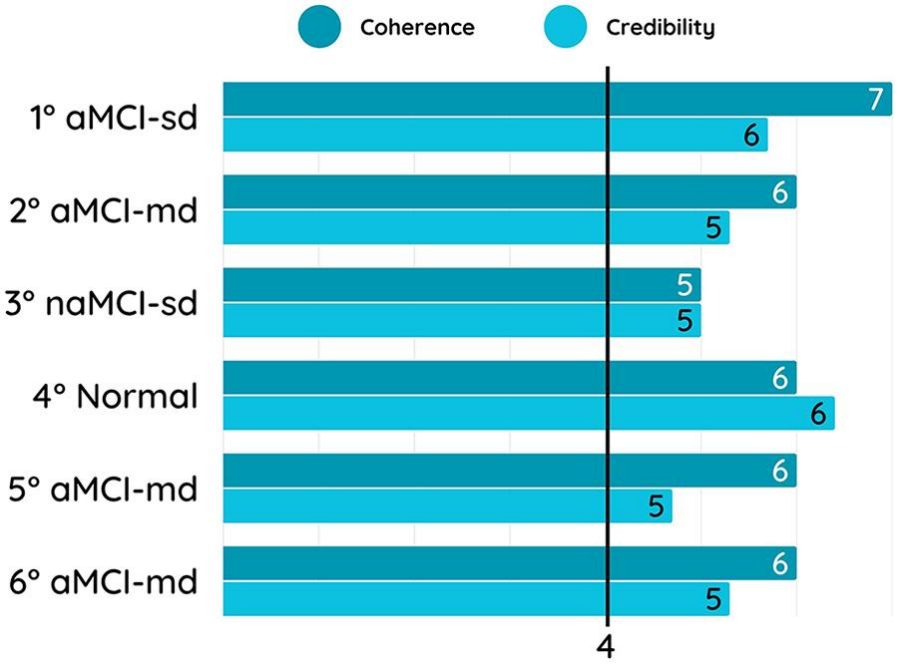
Average coherence and credibility scores for each dummy user profile. The y-axis displays the dummy user profiles: 1° aMCI-sd, single-domain amnestic Mild Cognitive Impairment; 2° aMCI-md, multi-domain amnestic Mild Cognitive Impairment, memory plus executive/attention; 3° naMCI-sd, single-domain non-amnestic Mild Cognitive Impairment, executive/attention; 4° Normal, cognitively unimpaired profile; 5° naMCI-md, multi-domain non-amnestic Mild Cognitive Impairment, memory plus language; 6°naMCI-md, multi-domain non-amnestic Mild Cognitive Impairment, memory plus visuo-spatial. The x-axis reports the mean coherence and credibility scores, assessed using a 7-point Likert scale, ranging from 0 (very low) to 7 (very high). Scores represent the average ratings provided by internal and external experts for each dummy user profile.

The accuracy in correctly identifying the representativeness of dummy users’ profiles was above 75% for both internal and external experts (Figure 1). At the post-test qualitative interviews, experts reported some challenges in the classification due to the different options provided by the questionnaire. Coherence and credibility of dummy user profiles was overall good (i.e., scores ≥4 at the 7-point Likert questionnaire) for both internal and external experts (Figure 2). Post-test qualitative interviews revealed that the slightly lower credibility scores were due to low trustiness in simulations, being the experts aware of the fact that they were asked to judge dummy users.

## 4 Discussion

In this study, we describe a simulation approach for the validation of a teleneuropsychology software as SaMD developed for remote cognitive testing in memory clinics [i.e., Tenepsia® (9)]. The use of such approaches is growing in interest in the digital health field (29). As proved by a recent consensus study involving regulators, industry and academic experts (29), this well-recognized step within the MDR 2017/745 pathway is a valid method to provide early-stage evidence of SaMD technical performance and support pre-clinical assessment before the authorization of clinical investigations and real-patient testing. In teleneuropsychology, it may allow the verification of scoring logic, consistency of software performance metrics with real-world use, and functional robustness under controlled conditions, without exposing patients to risks of an uncertified MD (28). Therefore, such preliminary MDR approaches enrich, rather than replace, clinical validation by demonstrating that the system can reproduce and discriminate heterogeneous cognitive profiles.

Our results indicate that the scores simulated in the platform are largely consistent with profiles of potential patients in real-world scenarios. The use of simulations with dummy users provided indeed good accuracy, and coherent and credible simulated cognitive profiles supporting the validation of the platform. Experts confirmed the adequacy of the work done in terms of simulation, through the accurate identification of profiles and their positive evaluation. The digital tests integrated into the platform effectively provide comparable information with their paper-and-pencil counterparts, ensuring equivalent results. Simulated avatars have been equally validated by internal experts from the study group and external clinical experts not involved in the project, demonstrating good consistency and credibility in both groups.

This approach have many advantages in teleneuropsychology for both final users, i.e., patients and clinicians. First, it avoids ethical concerns related to real-life testing of the digital solution on cognitive patients, such as the potential psychological burden, discomfort, or stress that the neuropsychological assessment may cause, especially in vulnerable populations like individuals with dementia or cognitive decline (8). Additionally, it avoids exposing human participants to unproven technologies, thus minimizing the risks associated with early-stage testing. The simulated approach also offers a time-saving and controlled environment for technical testing, free from the unpredictable factors that can arise in clinical settings. This is especially relevant in dementia, where cognitive deficits can impact reliability of collected data (30). One of the main challenges in testing novel digital solutions is the variability in patient responses and clinical settings. Dummy patients ensure a reproducible and standardized setting environment, which enhances the reliability of the data collected. This consistency is crucial for regulatory bodies when assessing the validity and safety of a new device. In addition, synthetic data are generated reducing expenses related to data acquisition, storage, and cleaning and enable the creation of reliable datasets that preserve statistical characteristics without compromising sensitive information. Finally, the controlled environment of simulated approaches also provides a consistent baseline for testing different MDs, allowing to pinpoint and rectify problems more effectively before moving on to real-life trials. This accelerated process can lead to quicker innovations and faster market entry for new digital devices, ultimately benefiting both patients and clinicians. Moreover, the availability of certified digital platforms may also have a significant impact in the neurorehabilitation setting, enabling continuous cognitive monitoring and the personalization of remote cognitive training programs, which are particularly valuable in long-term care pathways.

Some limitations of the current study should be acknowledged. According to the feedback provided by the experts, the lack of qualitative information on medical history and of observation of patient's attitude during the neuropsychological examination may affect the credibility of simulated performances. Previous training on questionnaire evaluation is also to recommend to improve the reliability of experts’ evaluations. Future validation studies are also needed to evaluate the consistency of our anecdotal findings, address diagnostic comparability across different administration settings (hospital- vs. home-based), and test usability in the target population.

In conclusion, simulation approaches represent a promising avenue for ensuring acceleration of digital innovation in clinical neuropsychology saving costs of direct comparison with real-life standard of care. While direct comparison with the standard of care remains a critical component of MD validation, simulated approaches with dummy users provide a complementary pathway addressing ethical, financial, and logistical challenges. This approach may also serve as valuable tool for training and education. Neuropsychologists can use simulations to become familiar with new devices and procedures, ensuring a smoother transition from development to clinical practice. This dual benefit enhances both the validation process and the practical implementation of new technologies in teleneuropsychology. Digital platforms for diagnosing and monitoring cognitive disorders will certainly contribute to a transition towards a more sustainable and equitable future, especially for the most fragile segments of the population.

## Data Availability

The original contributions presented in the study are included in the article/Supplementary Material, further inquiries can be directed to the corresponding author.
