# Supplementary material for: Accelerating digital innovation in clinical neuropsychology: simulation approach to support medical device certification
Source: Front Digit Health. 2026 Feb 12;7:1646694. doi: 10.3389/fdgth.2025.1646694 (PMC12936993; doi:10.3389/fdgth.2025.1646694)
Supplement: Supplementary file 1 [file Datasheet1.pdf]

Supplementary Material

Supplementary Table S1. *Ad hoc* developed questionnaire to assess representativeness, coherence and credibility of each dummy user profile

Domain	Question	Answer Options
Representativeness	Which of the following cognitive profiles is represented by the dummy user number # ?	Normal cognitive profile<br>aMCI-sd [memory impairment]<br>naMCI-sd [visuo-spatial impairment]<br>naMCI-sd [executive/attention impairment]<br>naMCI-sd [language impairment]<br>aMCI-md [memory, executive/attention & language impairments]<br>aMCI-md [visuo-spatial, memory & social cognition impairments]<br>aMCI-md [memory & social cognition impairments]<br>aMCI-md [memory & executive/attention impairments]<br>aMCI-md [memory & visuo-spatial impairments]<br>aMCI-md [memory & language impairments]<br>naMCI-md [language & visuo-spatial impairments]<br>naMCI-md [executive/attention & language impairments]
Coherence	How coherent is the cognitive profile of the dummy user number # ?	7-point Likert Scale from 1 (not at all coherent) to 7 (very coherent)
Credibility	How credible is the cognitive profile of the dummy user number # ?	7-point Likert Scale from 1 (not at all coherent) to 7 (very coherent)

aMCI: amnestic mild cognitive impairment; naMCI: non amnestic mild cognitive impairment; sd: single-domain; md: multi-domain

Supplementary Table S2. Demographic features and simulated scores of dummy users

Dummy Patient	Age	Education	Sex	Complex Figure Copy	Naming	Fluency (P/F)	Fluency (S/L)	Fluency (animals/fruits)	Fluency (furniture /vehicles)	Picture Description	Complex Figure Delayed Recall	Spatial Supraspan	FCSRT immediate recall	FCSRT Total Immediate Recall	FCSR Delayed Recall	FCSRT Total Delayed Recall	Stroop Time	Stroop Errors	Digit Symbol	Copy of Pentagons	Clock Drawing	Emotion Recognition
#1 aMCI-sd	73	5	M	31.5	14/16	14	11	16	9	12s<br>19v<br>30n	3.5	3.66	17	24	6	8	13.3	0	28	14	12	25
#2 aMCI-md	73	5	M	31.5	14/16	14	11	16	9	12s<br>19v<br>30n	3.5	3.66	17	24	6	8	29.7	4	11	14	12	25
#3 naMCI-sd	73	5	M	31.5	14/16	14	14	13	11	12s<br>19v<br>30n	15	19.46	31	16	12	4	31.4	5	9	14	12	25
#4 Normal	73	5	F	31.5	14/16	14	11	16	9	12s<br>19v<br>30n	15	19.46	31	16	12	4	14.9	0	28	14	12	25
#5 aMCI-md	73	5	F	31.5	11/16	4	4	8	3	12s<br>19v<br>20n	3.5	3.66	17	24	6	8	14.5	0	28	14	12	25
#6 aMCI-md	73	5	F	23	14/16	14	11	16	9	12s<br>19v<br>20n	3.5	3.66	17	24	6	8	12.8	0	28	10	6	25

aMCI: amnestic mild cognitive impairment; naMCI: non amnestic mild cognitive impairment; sd: single domain; md: multi domain; FCSRT: Free and Cued Selective Reminding Test; s: sentences; v: verbs; n: nouns
